# Periprosthetic fracture in the setting of periprosthetic joint infection of the hip: evaluation and management of a single-centre cohort with forty six patients

**DOI:** 10.1007/s00264-025-06636-8

**Published:** 2025-08-20

**Authors:** Markus Jaschke, Stavros Goumenos, Carsten Perka, Ulrich Stöckle, Andrej Trampuz, Sebastian Meller

**Affiliations:** Charité-Universitätsmedizin Berlin, Corporate Member of Freie Universität Berlin, Humboldt-Universität zu Berlin and Berlin Institute of Health, Center for Musculoskeletal Surgery (CMSC), Berlin, Germany, Berlin, Germany

**Keywords:** Periprosthetic joint infection, Periprosthetic fracture, Outcome, Risk factors

## Abstract

**Aims:**

Periprosthetic joint infection (PJI) and periprosthetic fracture (PPF) belong to the most devastating and complex complications following total hip arthroplasty, especially when both occur simultaneously. The aims of this study were (1) to evaluate the clinical outcome of PJI treatment in the setting of concomitant PPF and (2) to determine risk factors for reinfection.

**Methods:**

This study retrospectively analysed PPF occurring during the treatment of PJI in total hip arthroplasty (THA). In total 46 THA were included with concomitant PJI and PPF, of which 28 fractures occurred during the explantation and 13 fractures occurred during the interval between explantation and reimplantation. The median follow-up period was 66 months (range: 24–90) and minimum follow up of at least two years. Patients without infection recurrence after their surgical management were considered to have a successful clinical outcome. Reinfections rates were estimated through Kaplan-Meier curve and potential risk factors for reinfection were analysed with univariate non-parametric tests. Multivariate Cox hazard regression was used in order to account for confounders.

**Results:**

Overall, the success rate in our cohort was 71%. The overall mortality rate at follow-up was 22%. The estimated cumulative reinfection-free survival was 68% (95% CI = 80.9–50.5%) with a reinfection risk of 20% (95% CI = 10.3%-35.3%) in the first 12 months. Two patients (4.8%) had a re-fracture and another two (4.8%) had a recurrent hip dislocation that needed surgical intervention. High virulence pathogens (*p* = 0.038), difficult-to-treat pathogens (*p* = 0.016) and number of debridement during the interval (*p* = 0.043) were significant risk factors for reinfections. Difficult-to-treat pathogens were shown to be independent risk factors (*p* = 0.040).

**Conclusion:**

The reinfection rate of concomitant PJI and PPF is high and target treatment should be achieved. Significant risk factors for reinfection were presence of difficult-to-treat or highly virulent pathogens and increased number of debridement between explantation and reimplantation.

## Introduction

Periprosthetic joint infections (PJI) and periprosthetic fractures (PPF) represent two of the most severe complications following joint replacement. While each complication poses significant challenges to manage individually, their simultaneous occurrence makes treatment exceptionally complex, occasionally reaching a point where limb salvage procedures become exceedingly difficult [1].

The fundamental principle of treating chronic PJI is prosthesis exchange, as microorganisms survive on the implant surface by forming biofilms that shield them from local host immune defences. On the other hand, PPF is usually treated by stabilisation of the fracture via internal fixation with another implant. Concomitant occurrence of PJI and PPF is therefore immensely troublesome as the treatment approach of each complication is fundamentally contradictory to the other [2, 3].

The therapeutic approach typically involves fracture stabilization, surgical debridement, and prolonged targeted antibiotic therapy, coordinated through multidisciplinary team consultations. However, this comprehensive treatment course can significantly impact patient morbidity and lead to markedly increased mortality in affected cohorts.

With the rising incidence of THA and the corresponding increase in PJI among an aging population, mortality becomes a critical concern. Mortality rates associated with PJ are alarmingly high, exceeding those of many common cancers. The 1-year mortality rate for PJI is approximately 5%, while the five year mortality rate can exceed 20% [4].

Since the infectious component is taking a major part in this condition, financial matter can be compared to PJI for this. Due to prolonged hospital stays, increased perioperative morbidity and higher frequencies of readmission, the medical costs of treatment of these patients are significantly higher than in primary THA or uncomplicated revision surgery. If current epidemiological trends persist as anticipated, the incidence of these complications—both as isolated events and in combination—will likely rise substantially in the coming decades, with a significant impact on our society and healthcare system [5, 6]. While large-scale studies remain limited, the most reliable evidence suggests an elevated risk of recurrent infection following revision THA for PFF, surpassing the risk observed in revisions performed for other indications.

The aims of this study were [1] to evaluate the clinical outcome of PJI treatment in the setting of concomitant PPF (primary endpoint) and [2] to determine risk factors for reinfection. Our hypothesis is a high reinfection rate after treatment of simultaneous PJI and PPF.

## Materials and methods

### Study design

This study was designed according Strengthening the Reporting of Observational studies in Epidemiology (STROBE).

We retrospectively analysed data from a prospective cohort of patients, with initiation of data collection in the year 2012, diagnosed with PJI and treated at a single academic reference centre specializing in septic musculoskeletal surgery with interdisciplinary specialists in microbiology, infectious disease, pathology, orthopaedic and trauma surgery. Data collection did not follow a pre-specific protocol for this study. Additionally, this study was prepared by two independent clinicians and afterwards the results were compared. After institutional board approval (EA4/040/14) data were collected from a digital database using ICD-10 identification for PJI and PPF. Patients meeting the inclusion criteria were contacted for follow-up evaluation in the rooms of the academic centre. Furthermore, data was extracted from the internal documentation. During the follow-ups, clinical evaluation of the wound occurred, lab values were taken and x-ray imaging of the PPF were taken.

Inclusion criteria were chronic PJIs of THA with concomitant, surgically managed PPF during the period of infection treatment between January 2013 and December 2022. The diagnosis of PJI was established by using modified criteria of the International Consensus on Musculoskeletal Infection (ICM 2013) [7]. All patients underwent surgical management of PJI with a two stage THA revision. The concomitant PPF was addressed as necessary through Open Reduction and Internal Fixation (ORIF) and revision arthroplasty procedures in the final reimplantation.

Initial exclusion criteria were patients under the age of 18 and acute or chronic PJIs not treated with two stage implant exchange. Furthermore, PPFs during reimplantation were excluded as at time of reimplantation the PJI is considered as eradicated.

Pathogens implicated in the primary infection of the prosthesis were divided into high virulence and low virulence. *Staphylococcus aureus*,* Streptococci*,* Enterococci*,* Gram negative bacteriae and Candida strains* were considered as high virulence, whereas *Coagoulase negative Staphylococci strains (CNS)*,* Cutibacterium* and anaerobes were considered as low virulence [8]. Furthermore, pathogens with reduced susceptibility to antibiofilm antibiotics and candida strains were characterised as difficult-to-treat (DTT) [9]. Samples with more than one pathogen were considered as polymicrobial.

Standard protocol of tissue sampling for microbiological culture consisted of 5 tissue samples from the deep surgical site in every surgery with additional sonication of the infected prosthesis. Additionally, one tissue sample adjacent to the implant was sent for histopathological examination.

Antimicrobial treatment was based on the pathogens’ susceptibility given by the antibiogram and in accordance with our infectious disease specialists’ consultation out of our multidisciplinary team. Our antimicrobial protocol briefly consisted of empiric IV administration of ampicillin/sulbactam + vancomycin until the culture results were obtained and then a total duration of 12 weeks using rifampicin combination for Gram positive pathogens and ciprofloxacin combinations for Gram negative pathogens.

The minimum duration of follow-up was 24 months starting from the day of reimplantation. Routine follow up evaluations were scheduled at one, three, 12, and 24 months postoperatively. Reinfections were diagnosed and treated based on the same algorithm mentioned above and also in accordance with the Delphi criteria for PJI treatment failure [10].

Apart from reinfections, mortality-related-to-disease and other perioperative surgical complications were also documented. The main causes of mortality in our cohort were due to conditions related to the fracture or infection such as lack of mobilisation and its following complications in combination with advanced age and multimorbidity of the patients.

Patients without clinical signs and symptoms of infection recurrence including negative blood results after reimplantation were considered to have a successful clinical outcome. The same applied for deceased patients, who had no signs or symptoms of reinfection after reimplantation.

### Risk factors

The various risk factors related to PJI recurrence were divided into general, surgical and microbiological. General factors were basically patient demographics and included age, gender, BMI, American Society of Anaesthesiologists physical status classification (ASA score), Charlson comorbidity index (CCI) [11], clinical status of the host and grade of the affected extremity (McPherson score) [12] and number of prior septic and aseptic operations of the affected limb. Surgical factors were number of reoperations during the interval, interval length and Vancouver (UCCS) classification of PPF, Paprosky classification [13], surgery duration time, use of cemented or uncemented implants in the infected prosthesis or during the reimplantation and the material of the implants in use (metal or ceramic). Microbiological factors consisted of pathogens’ virulence grade (high or low), DTT organisms, polymicrobial infection and positive culture at time of reimplantation.

### Statistical analysis

Statistical analysis was performed using SPSS statistics v.23 (IBM, Chicago, IL). *P*-values < 0.05 were considered significant. Confidence intervals were set to 95% and all tests were two tailed. Continuous variables were expressed as median with interquartile range (IQR) and categorical variables were expressed as percentages. Revision surgery due to reinfection was analysed using Kaplan Meier survival curves. Follow up was measured from the first day of reimplantation until the day of reinfection or the last follow up. Potential risk factors were analysed using a nonparametric test for continuous (Wilcoxon-Mann-Whitney-Test) and for categorical variables (Chi-square-test). Potential risk factors found to be significant were further analysed for confounders using a multivariate Cox hazard regression model with hazard ratio (HR) and confidence interval (CI).

## Results

### Clinical outcome of PJI and PPF management (primary endpoint)

A total of 172 consecutive patients were identified, of which 85 patients were excluded after accounting for duplicates, location of fracture compared to infection site and PPF preceding the PJI or occurring not during the period of diagnosis and treatment of PJI. Another 6 patients were lost before their follow-up was completed. Furthermore, fractures during reimplantation were excluded as pathogen eradication is considered at times of reimplantation.

**Fig. 1:**
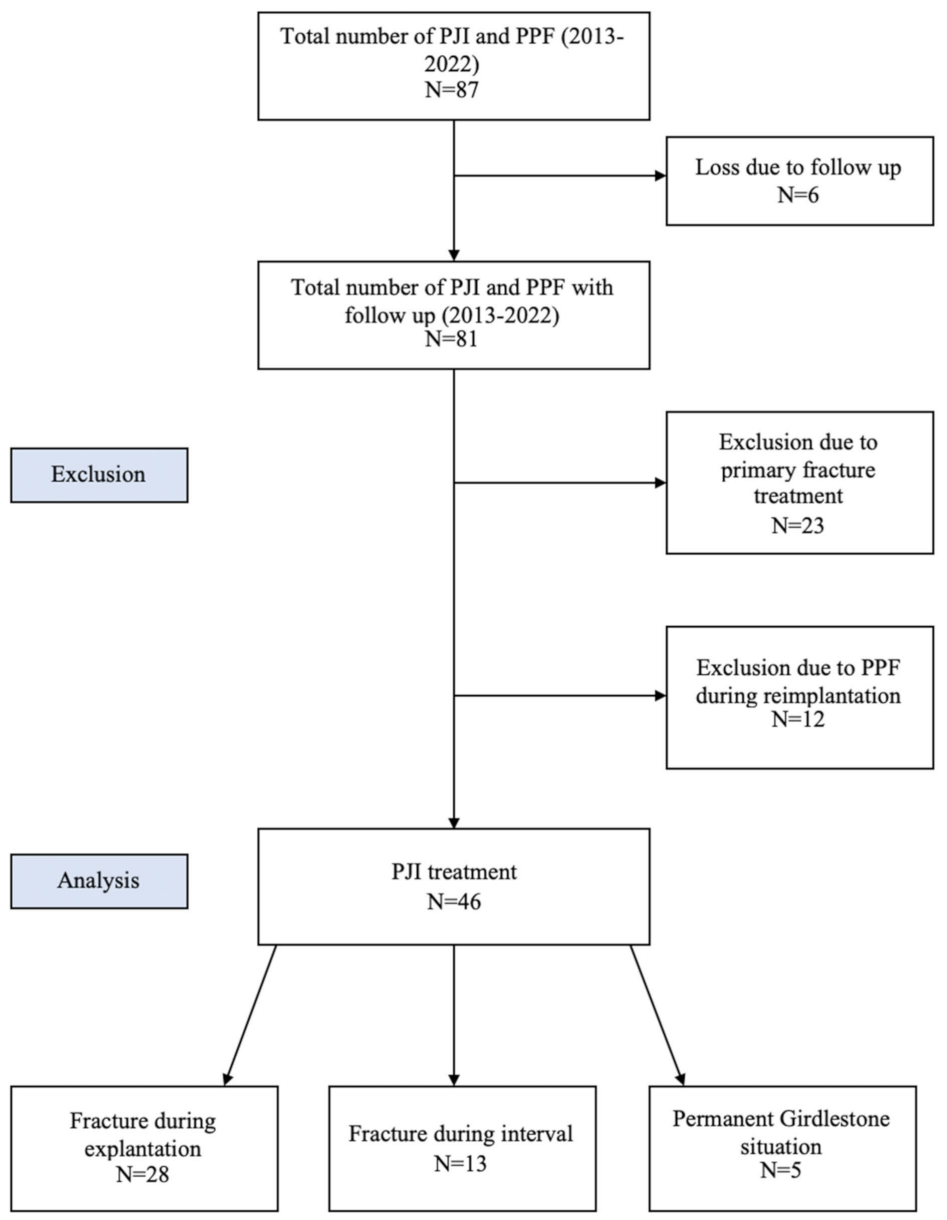
Data retrieval from patients with concomitant PJI and PPF operated between 2013 and 2022

Of the 41 patients 15 (36.6%) were male and 26 (63.4%) were female with a median age of 75 (IQR: 62-80.5) and a median BMI of 27.6 (IQR: 24.5–32.6, range: 14.6–49.6, mean ± SD: 28.5 ± 6.3). The median length of interval was 9.1 weeks (IQR: 7.0-13.7). The median follow-up period was 66 months (IQR: 32.5–90).

All of the remaining 41 cases with PPF during treatment for their PJI were included in the quantitative analysis, 28 (68.3%) of which occurred during explantation and 13 (31.7%) during the interval between the first and the second stage of treatment (Fig. 1).

The majority of fractures were stabilized using cerclage techniques, with plate osteosynthesis employed in select cases. Specifically, 38 of the 46 patients underwent stabilization with cerclages, while plate osteosynthesis was utilized in three cases. Regarding the timing of fracture occurrence, 28 fractures were noted during explantation, whereas 13 occurred during the interval between explantation and reimplantation. Fracture occurrence was distributed as follows: 28 fractures occurred during explantation and 13 during the interval between explantation and reimplantation. Lastly, five patients remained in a resection arthroplasty state (Girdlestone situation) due to their reduced general health. A reinfection was not observed in these patients at time of follow up. A quantitative analysis was therefore not performed with patients remaining in Girdlestone situation.

**Fig. 2:**
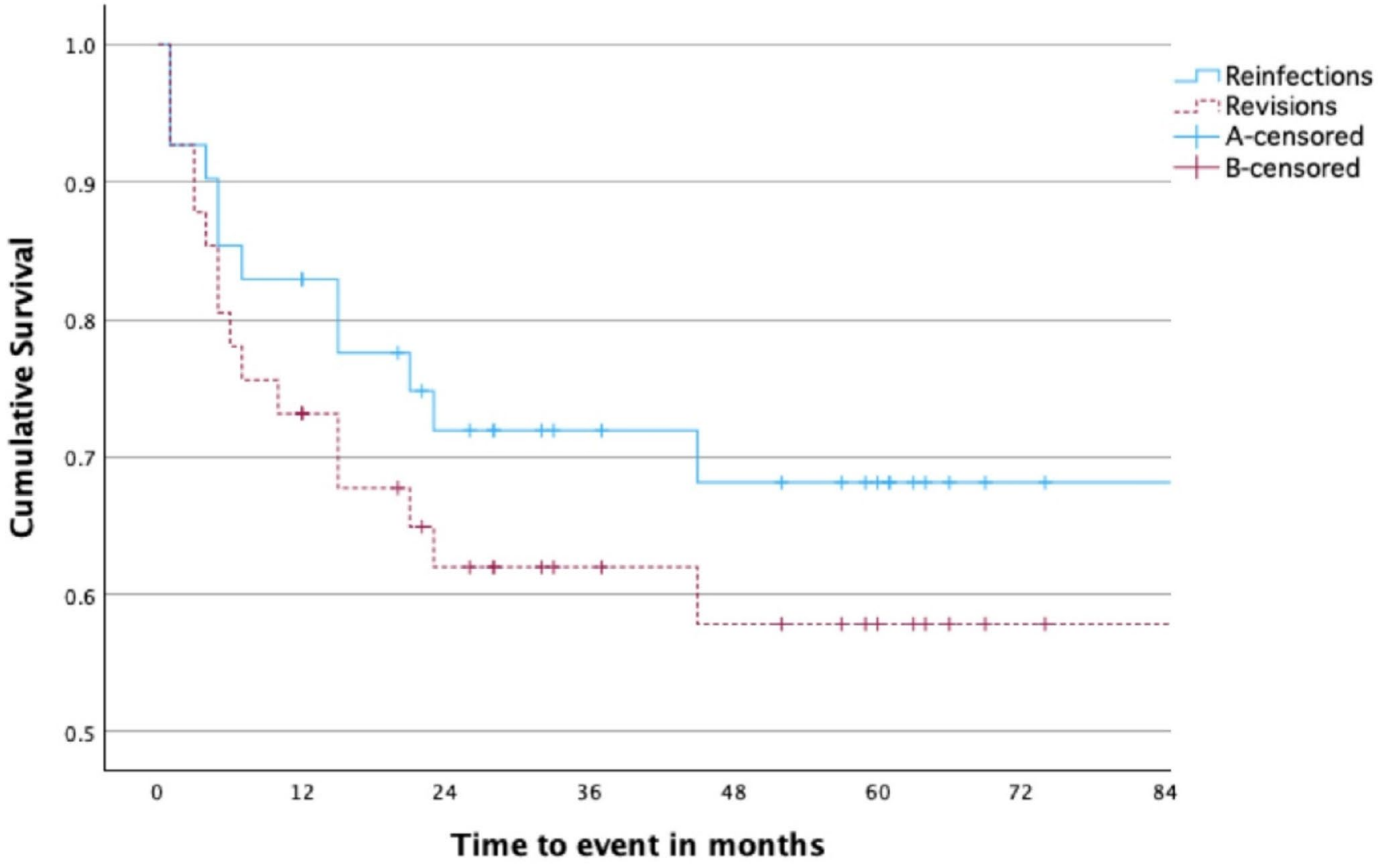
Kaplan-Meier survival curve of the risk of reinfection (red) and revision (blue) after reimplantation of treated PJI with following PPF

Overall, the success rate in our cohort was 70.8%. Of the 41 patients 12 had a revision surgery due to reinfection. The estimated cumulative reinfection-free survival was 68.3% (95% CI = 80.9–50.5%) with a reinfection risk of 17.1% (95% CI = 10.7%-32.5%) in the first six months, 19.5% (95% CI = 10.3%-35.3%) in the first 12 months and 27.9% (95% CI = 16.4%-44.8%) in the first 24 months after Kaplan-Meier analysis (Fig. 2).

Overall mortality was at 22% at time of follow up of which none died with signs of FRI or PJI recurrence. In total, the mortality related to the condition within the first year was 15%.

Of the 41 cases, two cases appeared with a re-fracture and two more had an aseptic hip dislocation that needed surgical intervention. The first re-fracture case was treated with cerclages while the second re-fracture case was treated with an additional plate and cerclages. For both patients with hip dislocation a closed reduction was attempted twice, but finally they underwent open reduction with revision of their acetabular component. Lastly one patient needed an evacuation of a surgical site haematoma two weeks after their reimplantation.

The cumulative probability of PJI recurrence in our cohort was 17.1% (95% CI = 10.7%-32.5%), 19.5% (95% CI = 10.3%-35.3%) and 27.9% (95% CI = 16.4%-44.8%) for the first six, 12 and 24 months after the reimplantation surgery, respectively. The estimated cumulative reinfection-free survival was 68.3% (95% CI = 80.9–50.5%). All reinfections occurred within the first 45 months postoperatively with a median follow up time of 66 months with a range between 12 months and 106 months. The cumulative probability of revision surgery in our cohort was 24.4% (95% CI = 12.1% − 40.6%), 29.3% (CI = 17.8% − 45.7%) and 37.8% (CI = 24.7% − 54.8%) for the first six, 12 and 24 months.

### General risk factors

The median values for ASA score and CCI were 3 (IQR: 2–3) and 5 (IQR: 4–6), respectively.

The median number of previous aseptic and septic operations were three (IQR: 2–4) and one (IQR: 0–2) respectively.

As for the McPherson clinical status score, 73% of the patients were host type B or C and 85% had a local soft tissue status score of 2 or 3.

The median number of days of hospitalisation were 19 days (IQR: 13.5–29.5) None of the analysed demographic parameters displayed a significant impact on the risk for reinfection after reimplantation. Lastly, non-unions were not observed. (Table 1).

**Table 1:** General risk factors for reinfection of concomitant PJI and PPF

	Success	Failure	Significance (2 sided)	Method
Age (years)	75 (IQR: 62–82)	69.5 (IQR: 61.5–78)	0.749	Mann-Whitney U
BMI (kg/m^2^)	27.5 (IQR: 24.5–30.5)	28.4 (IQR: 24.7–35.5)	0.549	Mann-Whitney U
Gender (M/F)	9 (31%)/20 (69%)	6 (50%)/6 (50%)	0.300	X^2^ (Fisher’s exact)
ASA score	2 (IQR: 2–3)	3 (IQR: 2–3)	0.772	Mann-Whitney U
CCI	5 (IQR: 3.5-7)	5 (IQR: 4-5.5)	0.575	Mann-Whitney U
Host type (A vs. B/C)	9 (31%)/20 (69%)	2 (17%)/10 (83%)	0.452	Chi square
Local soft tissue status (1/2/3)	5 (17%)/13 (45%)/11 (38%)	1 (8%)/7 (58%)/4 (33%)	0.661	Chi square
Prior 2 stage procedures (yes/no)	3 (10%)/26 (90%)	3 (25%)/9 (75%)	0.334	X^2^ (Fisher’s exact)
Prior septic surgeries	1 (IQR: 0–2)	1 (IQR: 0-1.5)	0.992	Mann-Whitney U
Prior surgeries	3 (IQR: 2–4)	3 (IQR: 2-4.5)	0.818	Mann-Whitney U

Continuous variables were given as median (IQR), categorical variables were given as number with percentage in parenthesis

### Surgical risk factors

Preoperatively 56% of the infected THAs had cement implanted without local antibiotics as structural support.

Concomitant PJI and PPF surgery time was on average 288 min ± 88 min with a median of 275 min (IQR: 231 –332 min, range: 131 min − 509 m). Additional interim debridements were significantly more frequent among the re-infected patients. Of the 13 fractures during the interval 12 were stabilised of which ten were stabilised with cerclages and two were stabilised with plates. Of the 19 fractures during explantation four fractures were classified as Vancouver A, tenwere classified as Vancouver B and five were classified as Vancouver C. The Paprosky bone defect classification did not show any significance in outcome. (Table 2).

**Table 2:** Surgical risk factor for reinfection in PJI with concomitant PPF

	Success	Failure	Significance (2 sided)	Method
Fracture (explantation/interval)	19 (66%)/10 (34%)	9 (75%)/3 (25%)	0.7186	X^2^ (Fisher’s exact)
Interval length (weeks)	10.5 ± 6.5 (6.9–12.9; 4.1–30.3)	(12.0 ± 5.1 (10.5; 8.3–13.7; 6–23)	0.204	Mann-Whitney U
Length of surgery in PJI and PPF	4 h 56 m ± 1 h 37 m (4 h 53 m; 3 h 48 m-5 h 56 m; 2 h 11 m-8 h 29 m)	4 h 29 m ± 59 m (4 h 24 m, 3 h 57 m-5 h 10 m; 2 h 58 m-6 h 35 m)	0.459	Mann-Whitney U
Cement infected hip prosthesis (yes/no)	15 (52%)/14 (48%)	8 (67%)/4 (33%)	1.0	X^2^ (Fisher’s exact)
More than one debridement during interval (yes/no)	5 (17%)/24 (83%)	7 (58%)/5 (42%)	**0.009**	X^2^ (Fisher’s exact)

Continuous variables were given as mean ± SD (median; IQR; range), categorical variables were given as number with percentage in parenthesis

### Microbiological risk factors

Overall, 23/41 infections (56.1%) were caused by high virulence pathogens and 18 (43.9%) by low virulence pathogens. Significant differences were found among re-infected and cured patients in terms of the virulence grade of pathogens and DTT organisms. In total seven (17.1%) patients grew DTT pathogens in their cultures. Furthermore, seven (17.1%) patients had a polymicrobial infection and a high virulence pathogen was implicated in all of them. During reimplantation 13 patients had positive cultures, yet in three cases a contamination was suspected, as there were only 1/5 positive sample with CNS strains, which were not originally implicated as primary pathogens during the time of PJI diagnosis. Therefore, 10 (25%) patients were considered having true positive samples at time of reimplantation (Table 3).

**Table 3:** Microbiological risk factor for reinfection in PJI with concomitant PPF

	Success	Failure	Significance 2 sided	Method
Virulence grade (high/low)	13 (45%)/16 (55%)	10 (83%)/2 (17%)	**0.038**	X^2^ (Fisher’s exact)
DTT (yes/no)	2 (7%)/27 (93%)	5 (42%)/7 (58%)	**0.016**	X^2^ (Fisher’s exact)
Polymicrobial (yes/no)	5 (17%)/24 (83%)	3 (25%)/9 (75%)	0.672	X^2^ (Fisher’s exact)
Positive culture during reimplantation (yes/no)	7 (24%)/22 (76%)	6 (50%)/6 (50%)	0.154	X^2^ (Fisher’s exact)

Continuous variables were given as mean ± SD (median; IQR; range), categorical variables were given as number with percentage in parenthesis

The median Harris Hip Score in the successful cohort was 79 (IQR:72.5–81.5, range: 65–89) and 70,5 (IQR: 63.5–75.5, 59–79) in the cohort with reinfection. The statistical analysis showed a significance with a *p*-value of 0.001.

The majority of our cohort (75%) remained infection free until the last follow up.

### Independent risk factors

The multivariate Cox hazard regression analysis of the significant risk factors of the univariate analysis (more than one debridement, virulence grad and DTT) showed a significance in DTT as being an independent risk factor (HR = 3.84, CI = 1.06 to 13.84, *p* = 0.040). The virulence grade and having more than one debridement did not prove to be independent (HR = 3.55, CI = 0.69 to 18.17, *p* = 0.128 and HR = 2.24, CI = 0.58 to 8.68, *p* = 0.244).

## Discussion

Due to the vastly more complicated procedure of two major complications simultaneously with opposing treatment strategies, success rate for concomitant PJI and PPF are lower compared to studies with only PJI showing a success rate between 8% and 20%. The reinfection rate of PPF during PJI treatment is notably high at 29.2%, yet we consider this revision rate to be a reasonable benchmark, as comparable data at this scale is unavailable. Preliminary estimates to estimate the infectious revision rate can be estimated by the study of Müller et al. and Karczewski et al. with slightly lower revision rate of 12.5% and 22.2%, respectively [14, 15]. It is important to note that these studies involved smaller patient cohorts (*n* < 10), which limits their statistical reliability. Sassoon et al. concluded a similar infectious revision rate of 24% for concomitant PJI and PPF in knee arthroplasty [16, 17].

Periprosthetic joint infection and periprosthetic fracture are both rare complications in the context of prosthesis implantation, with their simultaneous occurrence being exceptionally uncommon. A review of the literature reveals very limited information on concomitant PJI and PPF, particularly not on the scale presented in this study. Due to the rarity of this condition, precise data is not available about the prevalence. Estimates suggest that PJI occurs in approximately 1%-2% of cases [18], while PPF incidences range between 0.5% and 3% [19]. Revision surgeries significantly increase the incidence of PPF, rising to 4%-11% in total hip arthroplasty (THA) and up to 30% in total knee arthroplasty (TKA) [20].

Furthermore, epidemiological development is difficult to predict as for PJI medical treatment gets more advanced but immunological compromises are common in older individuals [21]. Similar relationship can be argued in PPF as osteoporosis progresses with age and physical activity maintain high due to medical development. Additionally, the rising average age of the population and the increasing demand for endoprosthesis implantation contribute to an increased rate of periprosthetic infections and periprosthetic fractures.

The mortality rates in our cohort are similar to those reported in the literature for periprosthetic joint infections, yet in our cohort we present additional periprosthetic fractures [4]. While the mortality was not directly attributable to PJI or PPF, we suspect an indirect correlation related to immobilization and its subsequent complications.

Most importantly we were able to show significances in number of debridement during the interval, DTT microorganisms and virulence grade of the pathogen. Higher rates of DTT pathogens seem to be more common in concomitant PJI and PPF as Karczewski was able to demonstrate [15]. Additionally, our results highlight that antibiofilm antibiotic resistance represents a significant risk factor, particularly in cases with reinfections. Microbiological risk factors emerged as critical determinants of treatment success or failure, likely due to the fracture management process. As a fracture is treated, foreign materials in form of cerclages or plates were implanted leading to risk of biofilm formation. This either was shown by pathogen culture analysis or manifested as additional debridement during the interval as symptoms of infection persisted due to implantation of foreign material. However, the precise connection between microbiological risk factors and fractures remain uncertain.

Furthermore, multivariable analysis showed having none or one significant risk factor gives quite promising results of infection-free survival 91% and 81%, respectively. In contrast, successful reimplantation rates dropped dramatically, with infection-free survival falling below 25% in patients with two or more significant risk factors. Although the number of debridement was identified as a risk factor in our results, we argue that debridement reflects the complexity of the condition rather than being an inherent risk factor itself.

The assessment of pathogens during reimplantation was challenged by the risk of potential sample contamination. To address this, the same criteria used for intraoperative tissue sampling during reimplantation were applied, following the modified guidelines of the International Consensus on Musculoskeletal Infection (ICM) as stated in the methods section.

Measured risk factors such as the host type, comorbidities and previous hip surgeries did not show any significance in our study. Furthermore, the length of the interval between explantation and reimplantation, polymicrobial infection and positive cultures also did not show a significant outcome.

The main limitation of this study is the relatively small number of patients which complicates the significance and strength of the results. However, to our knowledge it is the biggest cohort study addressing concomitant PJI and PPF with long follow up and reinfection evaluation. In contrast, most studies in the literature are limited to case reports, small cohorts of fewer than ten patients, or studies lacking follow-up data. Although the small cohort limits the results, the strength of this study is on the account of the large cohort size compared to the literature. Furthermore, statistical inaccuracy or bias might occur due the statistically small cohort size. Moreover, due to the complexity of concomitant PJI and PPF interpretation of the results tend to be inaccurate as different risk factors or even unknown risk factors have a relevant role in outcome. Lastly, due to a loss of follow up survivorship bias might occur to a limited extend as possible a negative outcome can be the case with patients not emerge after contact.

The main aim of this article is to investigate the reinfection rate of PJI with concomitant PPF, which lead to the above-mentioned definition. Müller et al. and Karczewski et al. [14, 15] used a similar definition for the final outcome in their study, while van den Kieboom et al. [17] included reinfections and differentiated PPF treatment, yet we acknowledge other definitions of success and failure. Furthermore, the mortality rates of our study are comparable with the mortality rates in the Literature for PJI and for PJI with concomitant PPF [4, 14, 15, 17, 22, 23]. 

Statistically, reinfection rate of concomitant PJI and PPF is associated with high failure rate. It can be hypothesized that pathogen colonization on osteosynthesis materials plays a critical role in the persistence of infection. Sonication of cerclages and plates might provide further insights into this issue. However, false negative results may be obtained due to extensive antibiotic treatment during the interval as cerclages and plates are being removed at time of reimplantation. Due to major risk factors being microbiological factors leading the decision in failure the main focus in treatment should rather remain in eradication of the pathogen. Nonetheless, patients with a successful outcome seem to have an acceptable outcome compared to the literature.

This study serves as an initial milestone in evaluating the combined conditions of PJI and PPF. However, further research is essential to enhance the understanding of this topic. We also advocate for multi-centre studies to compile larger patient cohorts in order to determine more precisely the outcome of concomitant PJI and PPF.

## Conclusion

The reinfection rate in cases of concomitant periprosthetic joint infection (PJI) and periprosthetic fracture (PPF) in total hip arthroplasty (THA) is high, with an overall estimated cumulative infection-free survival of 68.8%. Significant risk factors for reinfection were microbiological origin, including difficult-to-treat (DTT) or high-virulent pathogens, as well as the number of debridement performed between explantation and reimplantation. Early identification and targeted management of these risk factors are of high importance, especially in patients with two or more significant risk factors. The management of this complex condition requires a highly specialized interdisciplinary approach, with a primary focus on limb salvation and functional recovery.

## Data Availability

No datasets were generated or analysed during the current study.
